# Reconstruction and seepage simulation of a coal pore-fracture network based on CT technology

**DOI:** 10.1371/journal.pone.0252277

**Published:** 2021-06-24

**Authors:** Deji Jing, Xiangxi Meng, Shaocheng Ge, Tian Zhang, Mingxing Ma, Linquan Tong

**Affiliations:** 1 College of Safety Science and Engineering, Liaoning Technical University, Fuxin, China; 2 Key Laboratory of Mine Thermodynamic Disaster and Control of Ministry of Education, Fuxin, China; 3 College of Safety and Emergency Management Engineering, Taiyuan, China; 4 National Center for Occupational Safety and Health, NHC, Beijing, China; China University of Mining and Technology, CHINA

## Abstract

The distribution of multiscale pores and fractures in coal and rock is an important basis for reflecting the capacity of fluid flow in coal seam seepage passages. Accurate extraction and qualitative and quantitative analysis of pore-fracture structures are helpful in revealing the flow characteristics of fluid in seepage channels. The relationship between pore and fracture connectivity can provide a scientific reference for optimizing coal seam water injection parameters. Therefore, to analyse the change in permeability caused by the variability in the coal pore-fracture network structure, a CT scanning technique was used to scan coal samples from the Leijia District, Fuxin. A total of 720 sets of original images were collected, a median filter was used to filter out the noise in the obtained images, and to form the basis of a model, the reconstruction and analysis of the three-dimensional pore-fracture morphology of coal samples were carried out. A pore-fracture network model of the coal body was extracted at different scales. Using the maximum sphere algorithm combined with the coordination number, the effect of different quantitative relationships between pore size and pore throat channel permeability was studied. Avizo software was used to simulate the flow path of fluid in the seepage channels. The change trend of the fluid velocity between different seepage channels was discussed. The results of the pore-fracture network models at different scales show that the pore-fracture structure is nonuniform and vertically connected, and the pores are connected at connecting points. The pore size distribution ranges from 104 μm to 9425 μm. The pore throat channel length distribution ranges from 4206 μm to 48073 μm. The size of the coordination number determines the connectivity and thus the porosity of the coal seam. The more connected pore channels there are, the larger the pore diameters and the stronger the percolation ability. During flow in the seepage channels of the coal, the velocity range is divided into a low-speed region, medium-speed region and high-speed region. The fluid seepage in the coal seam is driven by the following factors: pore connectivity > pore and pore throat dimensions > pore and pore throat structure distribution. Ultimately, the pore radius and pore connectivity directly affect the permeability of the coal seam.

## 1. Introduction

At present, water injection [1–4] into a coal seam is the most effective way to reduce dust but is not an ideal solution in the field, mainly because the pore-fracture structure of coal seams is very complex, and its morphological and structural characteristics determine the physical and chemical properties of the coal [5, 6]. The occurrence and flow characteristics of fluid in different coal seam structures vary [7–9]. How to reconstruct the pore-fracture structure in coal seams and determine the relationship between the connectivities of the pores and fractures is an important means to determine the seepage flow capacity of the coal [10–12]. Recently, CT technology has been used to study the pore-fracture structure of coal seams. This method adopts fault imaging technology [13, 14], which has many advantages, such as nondestructive 3D digitization and refinement. Nondestructive visual measurement of the pore-fracture structure in a coal seam can be realized, and a 3D network model of real pores can be obtained.

Many scholars have carried out a considerable amount of research on the process of CT scanning restoration and reconstruction. Researchers R.SH.MIKHALL [15] and others have calculated the volume surface and hydraulic radius of a micropore group by analysing a kind of silica gel pore. J. T. Fredrich et al [16] revealed the geometric complexity of pore spaces by imaging the pore structures of 3D geological materials using laser scanning confocal microscopy and 3D reconstruction. C. R. Clarkson [17] carried out low-pressure nitrogen adsorption and high-pressure mercury intrusion measurements on shale reservoir coal samples. The results showed that the specific surface area and pore volume results were quite different among the coal samples. Hiroshi Okabe et al [18] used two-dimensional rock flakes to describe the three-dimensional pore space of a rock, assuming isotropy in the reconstruction process, and obtained three-dimensional stereograms. Li et al [5] used CT scanning technology and electron microscope scanning technology, the fracture pore structure at different scales is studied, and the influence of pore characteristics on coal seam gas storage capacity is analyzed. Alexandra Roslin et al [19] used CT scanning technology, the influence of confining pressure on coal seam fracture is analyzed. The results show that confining pressure can inhibit coal structure to some extent. Zhang et al [20] results showed that the effect of fracturing on the horizontal and vertical direction of coal body is obtained by the method of fractal dimension counting. After analyzing the pore volume and roar length before and after fracturing, it is concluded that liquefaction fracturing can improve the permeability of coal body. S.M.Shah et al. [21] used lattice Boltzmann and pore network models to simulate single- and two-phase flow, analysed porosity results, predicted porosity, unidirectional permeability and multiple physical properties at different length scales, and improved the flow law from the pore scale to the core scale.

At present, most scholars mainly focus on CT scanning to restore the distribution of pore fissures in coal rock [22–24]. Few studies have been performed on the multiscale characterization and modelling of coal [25, 26], the seepage flow law of water in real pores and the dynamic seepage evolution mechanism [27–29]. Based on this work, the dynamic seepage process of coal seam water injection has been accurately described. This paper uses the high-precision CT scanner by Dandong Aolong Ray Co., Ltd., to scan the coal body, filter the interference noise in the original image by a median filter, and reconstruct the three-dimensional pore network model of the coal body by using Avizo numerical simulation software. The pore and fissure structures at different scales are extracted [30–32]. The maximum sphere algorithm and coordination number are used to analyse the relationship between the connectivities of pore and fissure groups at different scales [33–35], and the effects of different quantitative relationships between the pore size and pore throat channel permeability are studied. Avizo software is used to simulate the flow path of a fluid in the seepage channel [36–39], and the trend of the variation in fluid velocity between different seepage channels is discussed to explore the mechanism of the microseepage of fluid in a coal body and provide guidance for coal seam water injection and dust control measures [40–42].

## 2. Material and methods

### 2.1. Coal sample collection

**Fig 1:**
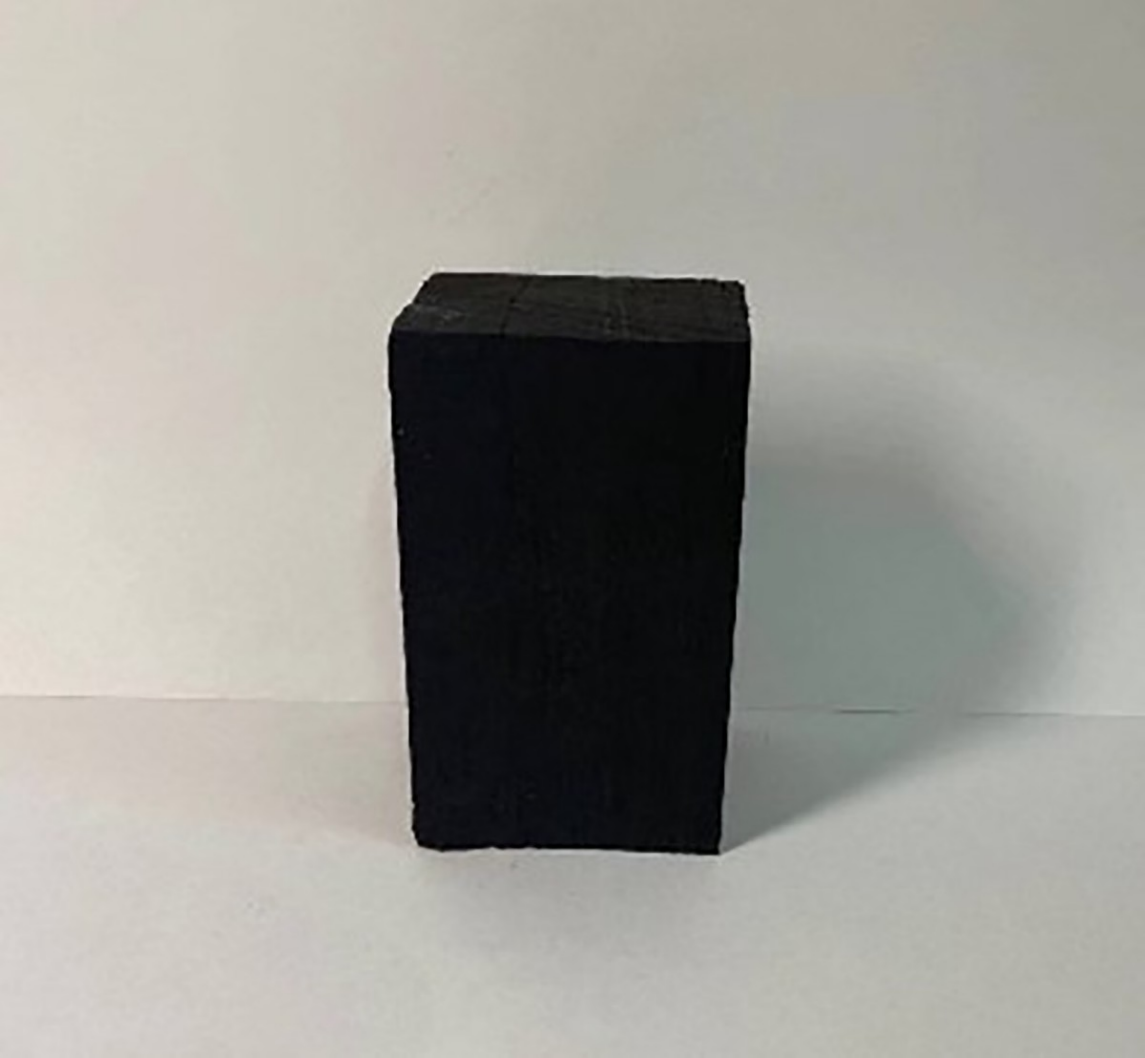
Experimental coal sample.

The coal was taken from the No. 4 coal seam in Leijia District of Fuxin City, and this coal is long-flame coal. According to the GB/T 482–2008 Chinese national standards for sampling, the coal specimen was cut into 5 cm×5 cm×10 cm cuboids by a standard coal sample cutting machine in the laboratory, and three standard coal samples were obtained. An SDLA618 proximate analysis of the coal was carried out by using an industrial analyser. The results are shown in Table 1. Experimental coal samples are shown in Fig 1.

**Table 1:** Industrial parameters of the coal sample.

Coal	Area	Degree of porosity	M_ad_%	A_ad_%	V_ad_%
long-flame coal	Fuxin Haizhou Mine	16.23%	6.75%	35.11%	77%

### 2.2. Experimental equipment and methods

The basic principle of CT detection technology is that the cross-section of the penetrating object absorbs the X-ray, and the detector receives the information of the X-ray passing through the section of the layer. When the uniform object is irradiated by X-ray penetration, the attenuation coefficient x of the object is exponential. The black and white colourscale in the grey image from the CT scanning process clearly characterize the density distribution of the object under detection, including the fissures, texture and other defects inside the object. The absorption ability of the object to the X-ray is also different, which is reflected by its attenuation coefficient. Thus, the pore structure of the coal body can be revealed without damage, and the coal sample can be further tested after CT scanning.

**Fig 2:**
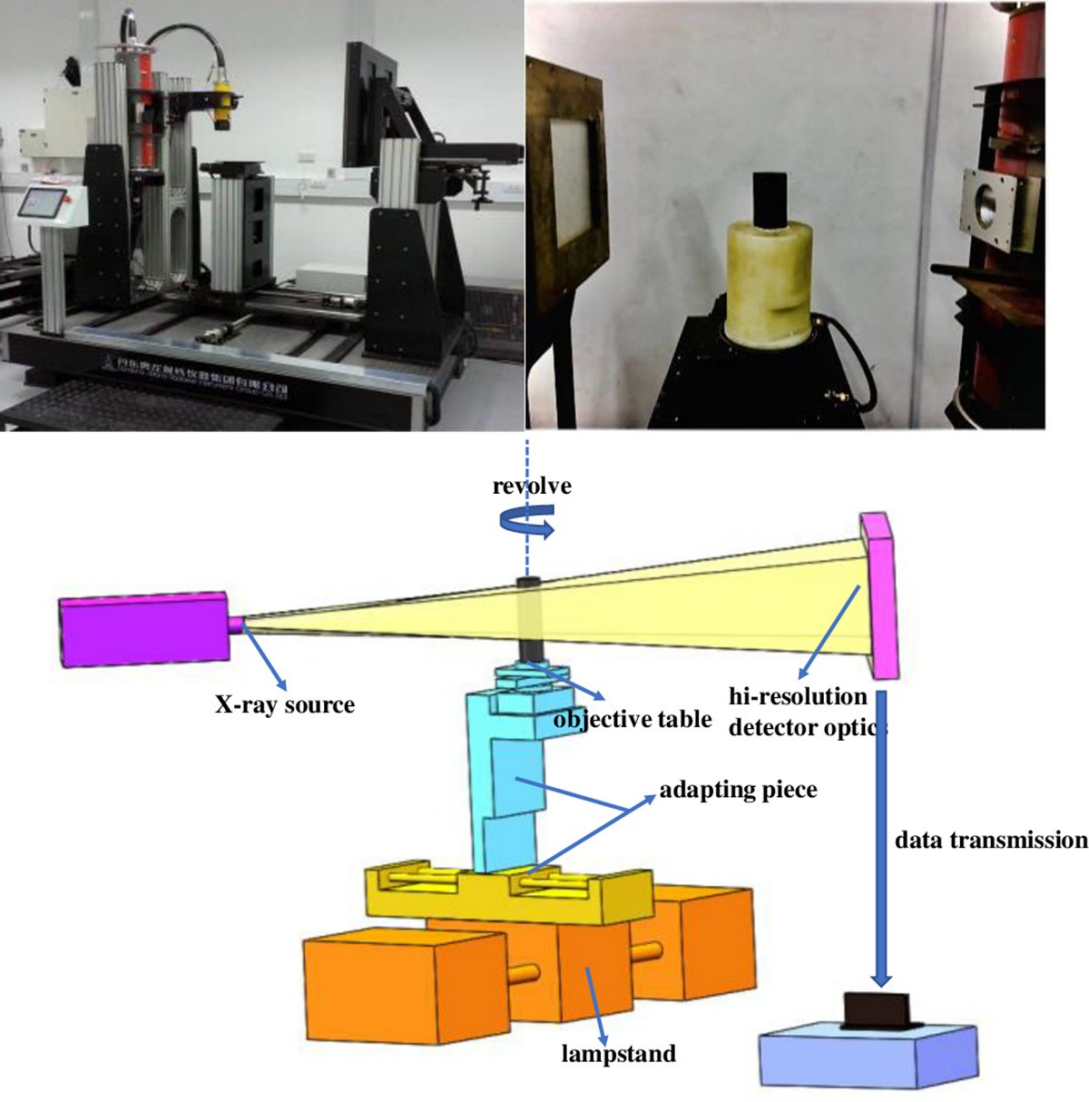
AL-CT-225 type CT scanning device.

As shown in Fig 2, using a Dandong Oron Ray Instrument Co., Ltd., AL-CT-225 industrial X-ray CT detection system, the coal sample was placed in the centre of the platform. A variety of detection methods, such as cone beam scanning and DR real-time imaging, were used. During 360° coal sample rotation, scan information about the defect locations and porosity was obtained. A total of 720 projection images were collected. The coal sample scan was performed at a radiation source voltage of 60 kV at a high temperature and constant pressure and a current of 240 μA. The minimum CT spatial resolution of the tomography was 6 μm, and the density resolution was 0.5, a maximum of 1024×1024 pixels.

### 2.3. Analysis of scan results

**Fig 3:**
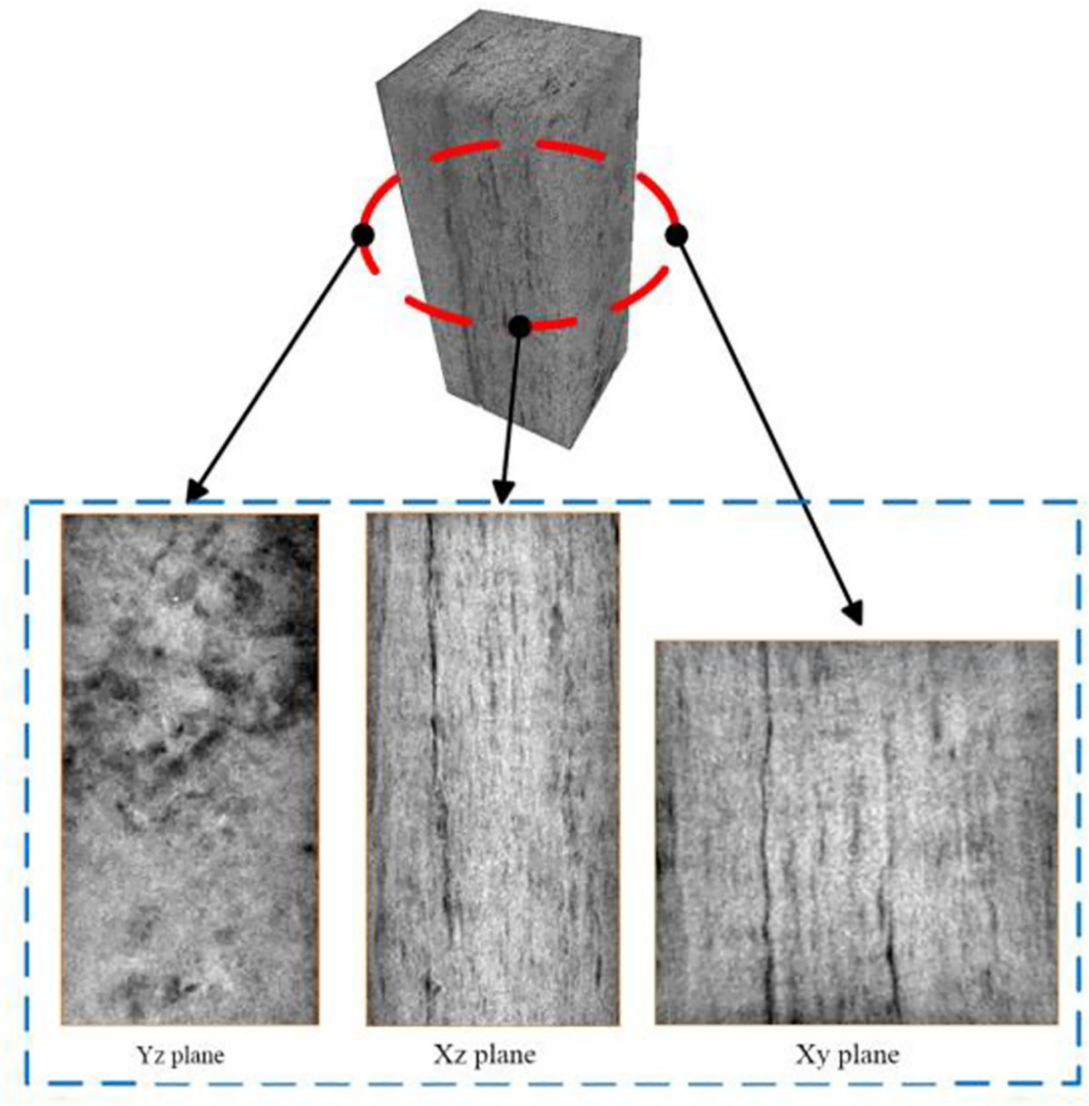
CT scanning of the pore-fracture structure of a coal sample.

An initial image of the coal sample is obtained by CT scanning technology, as shown in Fig 3. A group of slices in the yz, xz, and xy planes are selected. The black linear feature is a fissure in the coal sample, and the yz plane has a large number of pore-fracture groups. There are clearly fracture channels and pores visible in the coal matrix in the xz and xy planes. However, there is a considerable amount of noise in the original image, so it is necessary to select the appropriate filtering function to further process the image.

### 2.4. Median filtering of CT image noise

There is system noise in the original image obtained by X-ray CT scanning. The system noise is mainly due to the noise that inevitably arises in the process of data transmission. The noise is randomly distributed in the image. These noise points will increase or decrease the real pixel value of the image. Noise is often represented as an additional pixel position or block on the image. Therefore, the quality of the image is reduced, and the restoration, feature extraction and interpretation of the image are affected. In this paper, a median filter is selected to process the noise in the image. Processing the noise in the image can not only eliminate the noise in the image but also maintain the data of the original image. The grey value of the original image is not changed.

**Fig 4:**
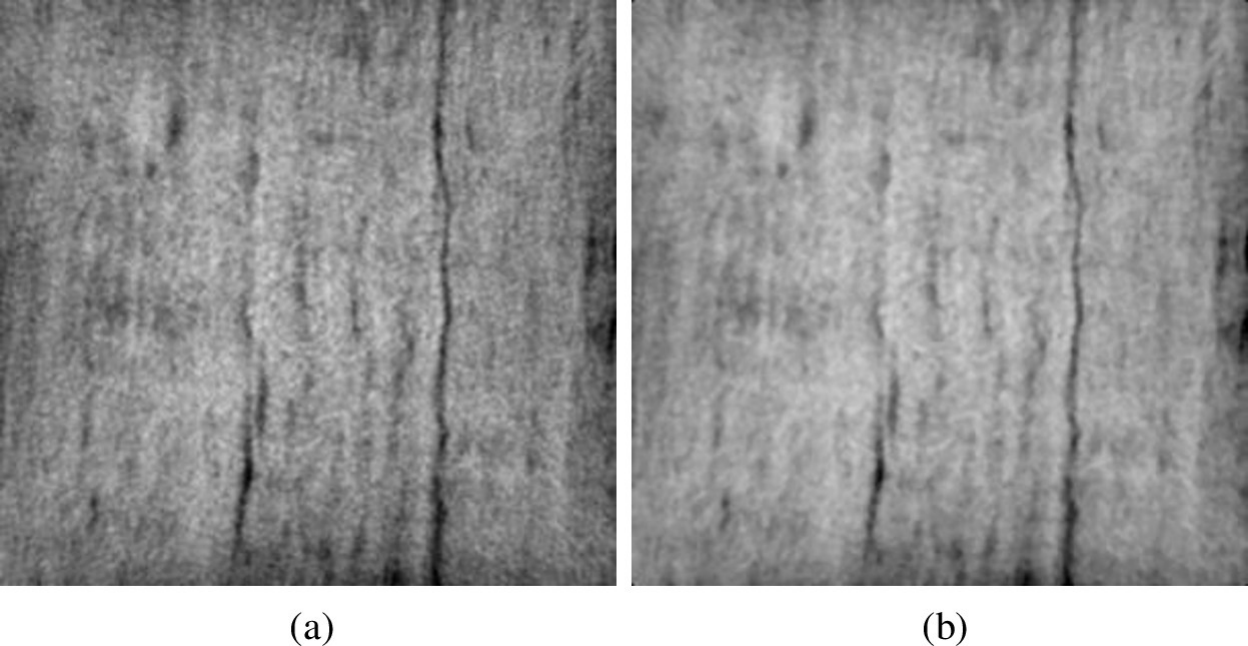
Image comparison before and after median filtering.

Fig 4(A) is the original image of a slice of the CT scan result, and Fig 4(B) is the image filtered by the median filter. The median filter is used to filter the noise points in the image, to protect the data of the original pore structure and clarify the edge position of the image clearly so that it is not blurred. The processed image can be used as the basis image for reconstructing the three-dimensional coal body structure.

## 3. Results and discussion

### 3.1. Comprehensive characterization of pore-fissure spaces at different scales

**Fig 5:**
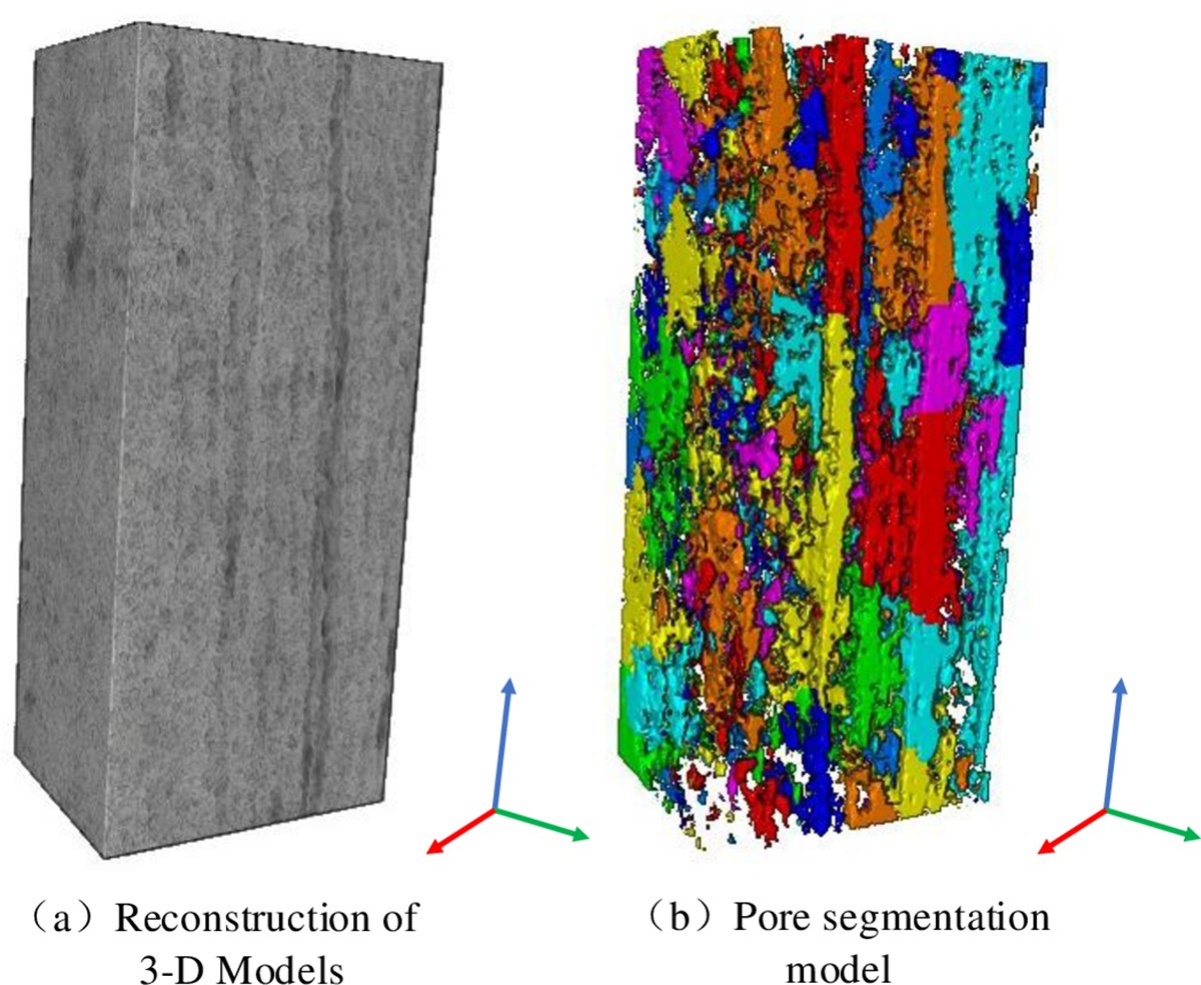
Comprehensive characterization of the pore-fracture network.

The coal images from the CT scanning reflect the pore-fissure characteristics of each layer. To analyse the three-dimensional pore-fissure distribution characteristics of the coal samples, a three-dimensional reconstruction of the coal samples is carried out. The internal structure at different scales is extracted and characterized. The two-dimensional CT slice after median filtering is used to construct the three-dimensional model of the coal sample by using the volume rending module in Avizo, as shown in Fig 5(A). The pore-fracture characteristics in the coal samples are extracted with the threshold segmentation module, The data parameters of pore volume 3d, pore radius and roar length of coal sample can be analyzed by using Label Ansysaily function in Avizo, and the pore model after segmentation of the coal matrix is accurate undamaged, as shown in Fig 5(B).

Fig 5 shows the comprehensive spatial characterization pattern of the pore fissures, in which the pore fissures of the coal sample result in massive, isolated pore blocks with a zonal vertical distribution. These blocks in the coal are distinguished by different colours, and the connected fissures are displayed in the same colour. The results of the quantitative analysis of the coal samples show that the total pore volume imaged is 3.6735×10^13^ μm^3^, the total pore area is 9.098×10^10^ μm^2^, there are a large number of micron-sized pores in the coal samples, and the coal samples show good three-dimensional uniformity in terms of the pore distribution.

**Fig 6:**
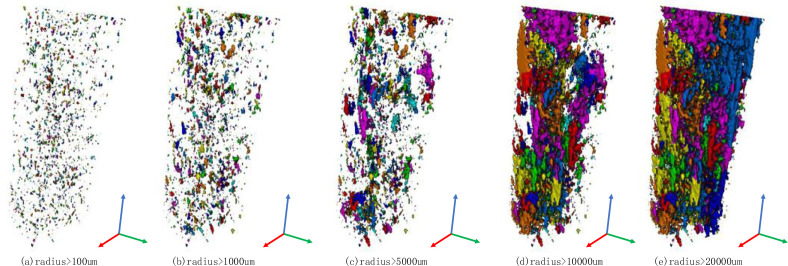
Distribution of pore radius at different scales.

As shown in the Fig 6, the three-dimensional spatial distribution of the coal pores at different scales is extracted. Fig 6(A) shows that there are a large number of very small pores; larger pores are added in Fig 6(B), and some connected pores are added in Fig 6(C). As the scale increases, the pore connectivity is gradually revealed. By extracting coal bodies at different scales, the distribution of different components at each scale can be clearly seen. The diagram shows that the pores of a sample will disform pore-fracture groups with increasing scale. This part of the connected pores will provide the main seepage channels for the coal body. In the process of seepage flow, hydraulic fractures communicate with connected pore groups.

### 3.2. Quantitative analysis of pores and pore throats

The diversity of the pore system of coal and rock depends on the pore structure distribution, which is reflected in the quantitative relationship among the pore size, pore volume area, pore throat channel radius and pore throat channel length. The distribution law of the pores and pore throat channels in coal samples is the key to determining the permeability of coal seams. The pore radius determines the connectivity between pore throat channels, and the radius and length of the pore throat channels directly determine the permeability of the coal seam.

**Fig 7:**
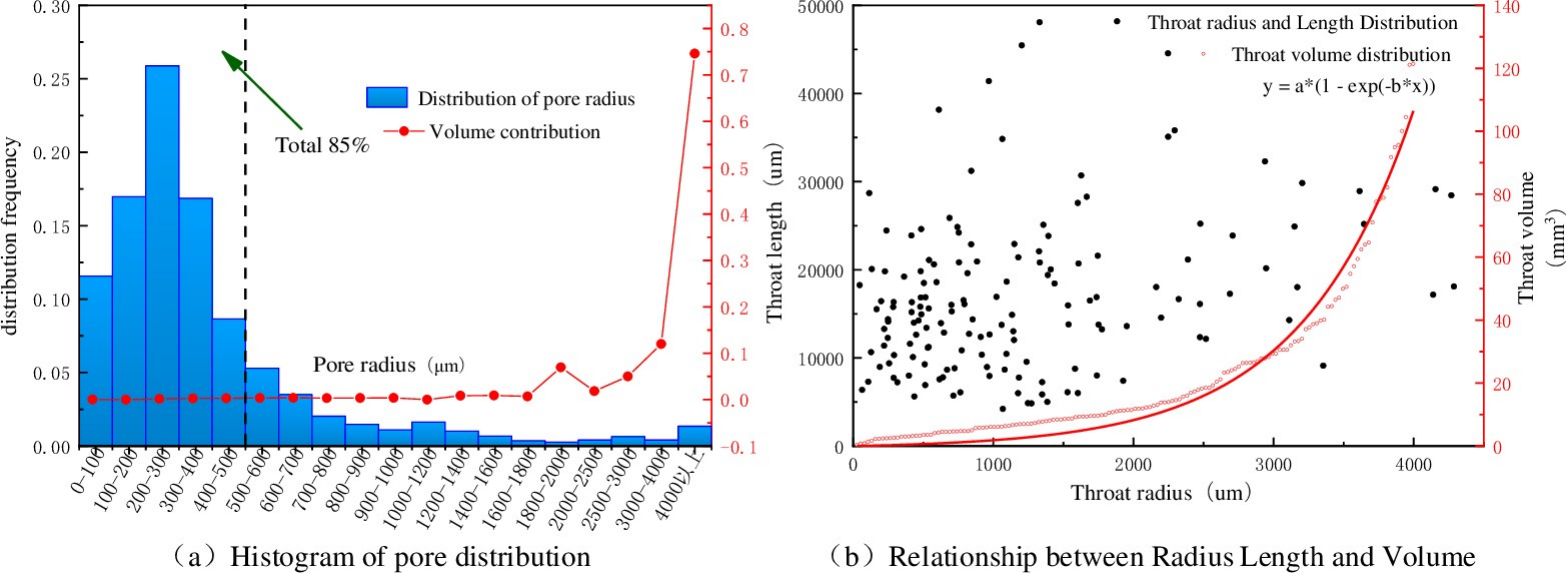
Distribution frequency of pore radius, pore throat radius and pore throat length.

As shown in Fig 7(A), the distributions of pore radius, volume contribution rate and pore throat radius and length of coal samples are given. Analysis of the data shows that the pore radii below 500 μm account for 85% of all the pores and that the pore radii greater than 500 μm account for 15% of all the pores. The coal samples have pores with sizes of 200–300 μm, and the pore throats in coal are well developed. The pores in a coal sample are well connected by the pore throats. The micron-sized pores in the coal samples are divided into micropores, transition pores and mesopores, defined in pore radius ranges of 0–500 μm, 500–2000 μm and 2000 μm, respectively. The three-dimensional spatial distribution constants of the micropores, transition pores and mesopores in the coal samples are shown in Fig 7. The pore size analysis of the coal sample data shows that the number of micropores, transition pores and mesopores is 2763, 598 and 96, respectively, the contribution of micropores and transition pores to the total pore volume is 0.78% and 11.21%, and mesopores account for 88.01% of the total pore volume. Despite the number of micropores and transition pores, due to their small sizes, micropores and transition pores contribute little to the connectivity of the fluid channels. The mesopores, due to their larger pore size, mainly provide the seepage passages. also show good connectivity in 3d visualization. Based on quantitative pore space analysis of coal samples containing micropores (79.92%) and transition pores (17.29%), the pore volume ratio is 11.99%, and the pore volume ratio also considering the moderately developed mesopores (2.79%) is 88.01%.

Fig 7(B) shows the scatter plot of the pore throat radius and pore throat length results of the coal samples and the corresponding law determined by exponential function fitting. The pore throat radii range from 46.29 to 4286.89 μm, and the lengths of the pore throats range from 4206.28 to 48073.5 μm. The volumes of the pore throats range from 0.4 to 121 mm^3^. From the diagram, most of the pore throat radii in the coal sample are in the range of 100–2000 μm, and the pore throat lengths are concentrated in the range of 5000–25000 μm. The results show that the distribution of the pore throat radius and length parameters in the coal samples are fairly uniform, and the pores are connected through the pore throat channels. Compared with the results of the complex 3D pore morphology of coal-like porous media, micron-scale pores are more developed in these coal samples. The three-dimensional pores are intricate, indicating that the macroscopic pores and microscopic cracks in coal samples have a complex spatial distribution and interconnectivity.

### 3.3. Connectivity analysis and network characteristics of a porous rock

To further explore the permeability of the coal samples, it is necessary to further extract the pore-fracture structure of the coal body, identify the pore position and form an equivalent pore network. The maximum sphere algorithm is generated by uniformly distributing spheres at the pore voxels in the pore space, one for every two voxels in each direction. The maximum ball algorithm is as follows:

$$EqDiameter=\sqrt[{3}]{\frac{6\times Volume3d}{\pi }}$$ (1)

EqDdiameter is the equivalent pore size, μm, and Volume3d is a single pore volume, μm^3^.

**Fig 8:**
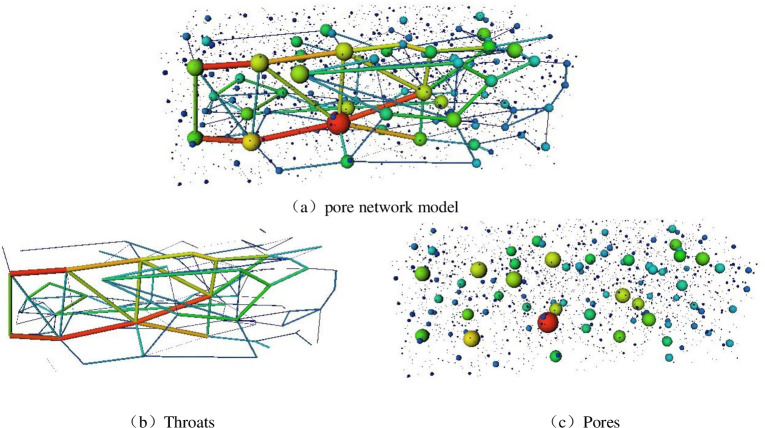
Pore network model.

In Fig 8, the spheres represent the radii of the pore throat channels. The coloured cylindrical connection represents the connectivity between the pore throat channels, that is, the permeability of the coal sample. The pore throat channel connectivity, pore throat channel length and pore throat channel radius directly affect the coal permeability. The coal-like pores and pore throats are extracted using the maximum sphere algorithm. The extracted pore network model has a total of 3458 pores, 151 pore throats and 93 connected pore channels (representing the coordination number). The branches and endpoints of the network in the figure are called pores, and the lines connecting pores are called pore throats. The maximum pore volume is 3.51×10^12^ μm^3^, and the corresponding pore area is 5.85×10^9^ μm^2^. The maximum capacity of the pore throat channel is 5.77×107 μm^2^, and the maximum length is 4286.89 μm. As shown in the pore network model in Fig 8, the coal samples contain a large number of very small pores, which are basically not connected with other pores. With increasing pore radius, the connected channels increase, and the permeability increases gradually. That is, the small cracks in the coal often appear as isolated points. Because they are not closely connected with the internal pores, the very small pores cannot be connected with the pore throat channels or contribute to the seepage. These isolated pore structures often do not have seepage characteristics. Because pores with larger pore sizes are fully developed and connected in the coal body, several complete seepage channels are formed with other pore channel tributaries, which plays a decisive role in the seepage flow capacity of the coal seam.

### 3.4. Matching analysis

**Fig 9:**
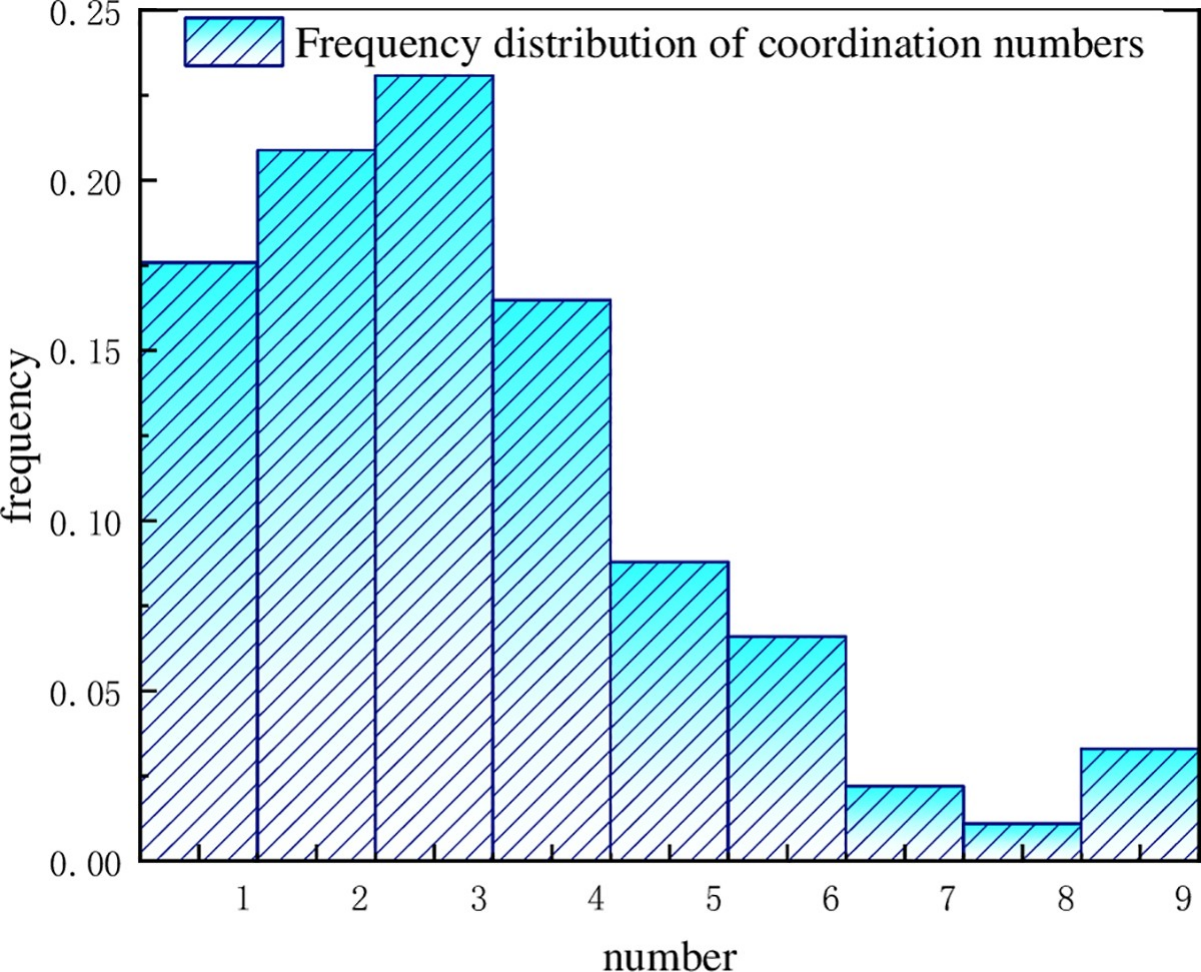
Histogram of distribution frequency of the coordination numbers.

The connectivity between pores directly determines the permeability of the coal seam. The coordination number reflects the connectivity between pores and pore throats, that is, if a pore is interlinked with pore throats. The pore structure parameters represented by the coordination numbers can reflect the spatial characteristics of the whole pore system and be used to analyse the pore system more accurately. Fig 9 shows the frequency histogram of the coordination number distribution.

Pores with a coordination number of zero can be regarded as isolated pores. This part of the pore characteristics often shows that the pore volume area is small, it is basically not connected with other fissures, and the contribution to the mass flow rate of the seepage channel is very small. The flow trajectory of the fluid in the coal body runs along the seepage channel, that is, the connected pores form the seepage zone region. In reality, this part of the pore needs other seepage channels to extend the pressure-driven seepage to the isolated pore area to form a complete seepage zone. For pores with more than one coordination number, a complete seepage channel is formed because of the connectivity with other pores. The seepage water moves along the seepage path, and the larger the coordination number is, the closer the connection and the greater the number of seepage channels in the coal. thus, the larger the coordination number is, the more seepage occurs in the coal, and the better the wettability of the coal samples.

### 3.5. Permeability simulation principle

The ability of porous media to allow single-phase fluid passage under a certain pressure gradient is defined in square metres, but square micrometres are more widely used in practice because 1 square micrometre is closer to 1 Darcy (d) and 1 d = 0.9869233 is the constant coefficient of the absolute permeability of a material according to Darcy’s law of fluid flow.


$$\frac{Q}{S}=-\frac{k}{\mu }\frac{△P}{L}$$ (2)

Q is the flow (m^3^/s) of fluid through the porous medium in a unit of time; S is the cross-sectional area of the porous medium (m^2^); is the absolute permeability (Pa.s); k is the dynamic viscosity coefficient of the fluid flow; △P is the pressure differential of the fluid in the medium (Pa); L is the length of the fluid passing through a porous medium (m).

$\frac{Q}{S}$ usually represents the flow velocity of fluid flowing through the surface of a porous medium and is also known as the Darcy velocity. When a porous medium is saturated with single-phase fluid, the permeability is called the absolute permeability, and when a porous medium is filled with a multiphase fluid, the permeability is called the relative permeability.

### 3.6. Permeability simulation results

For a further analysis of the percolation ability of the pore-connected channel, seepage simulation of the real pore network model is carried out, and seepage simulation of the coal sample is carried out in the x and z directions. The flow characteristics and transportation capacity of the fluid can be obtained by analysing the flow trajectory. The simulation software adopts AVIZO software, the model size is 5 cm×5cm×10cm, the inlet parameter is set to 1 MPa pressure, the outlet parameter is set to 0 MPa, the dynamic viscosity parameter of the fluid is set to 1.01×10^−3^ Pa·s.

**Fig 10:**
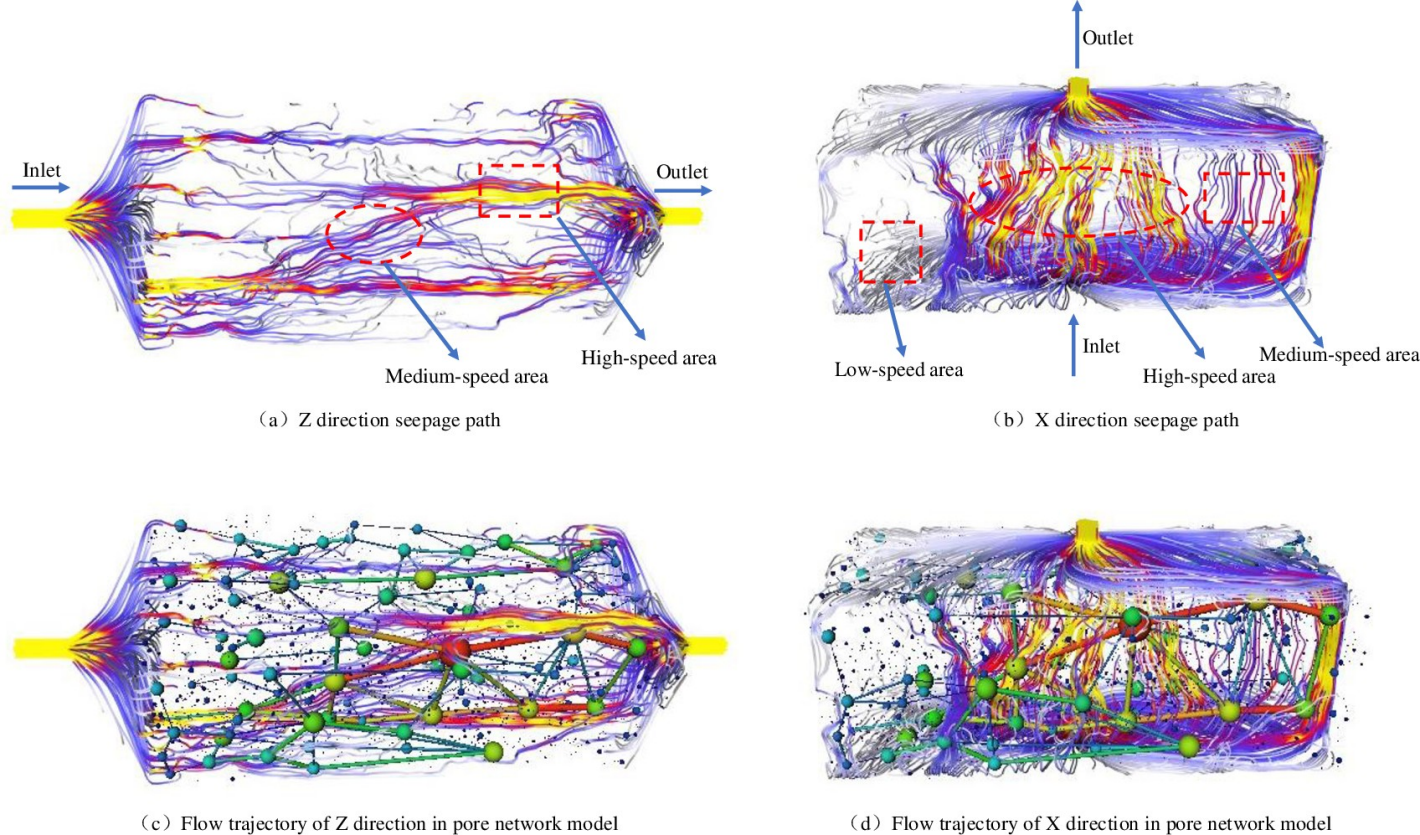
Flow trajectories in the x and z directions.

Fig 10(A) is a seepage trajectory simulation diagram for the z direction; the fluid direction is set to flow from left to right, a purple streamline represents the medium-velocity region of the fluid, and a yellow streamline represents the high-speed region of the fluid. The diagram shows that the high-speed region is the connected region among the pore throat channels, that is, the region in which each tributary connects to the others. The convergence of each branch leads to momentum superposition, which makes the velocity increase, and the fluid velocity is in the high-speed region. The medium-velocity region of fluid is mainly due to the poor development of pore fractures and the decrease in the pore throat radius in the coal sample, which leads to weak connectivity between the pore throat channels and the loss of some momentum when the fluid passes through the seepage channel. The fluid velocity is a medium velocity. To better observe the influence of pore connectivity and the length of the pore throat channel on the percolation ability, Fig 10(B) represents the pore throat channel connectivity and the percolation trajectory superposition diagram. The fluid velocity is faster in the superposition region of the large pores and seepage channels. In the narrow aperture area, the velocity is relatively slow. Fig 10(C) shows the trajectory map of the seepage in the x direction, and the grey streamlines correspond to the low-velocity zone of seepage. It can be clearly seen that the high-speed zone in the diagram is characterized by tight and large pores and that the region of smaller pores corresponds to the medium velocity region. The main reason for the low-speed region is that the pore throat channels are close to the outer wall of coal, and the pore connectivity is not good there. Fig 10(C) shows that when several seepage channels converge, the seepage channels are relatively flat, and the velocity is high.

In the flow of microscale pore structure, the whole structure of pore system is connected, but there are some isolated pore channels, which are basically zero. In the process of fluid migration in connected channels, the velocity change of fluid trajectory is more complex, which does not follow the linear and nonlinear relationship. First of all, the size of the roar volume is the most important factor to determine the seepage capacity, that is, the degree of pore connectivity. Secondly, the size of pore roar radius is a secondary factor to reflect the seepage capacity of coal seam. Finally, the distribution of pore roar structure is the final factor.

## 4. Conclusions

(1) The pore-fissure structures in coal samples are analysed qualitatively at different scales. The reconstructed 3D pore network models of the coal samples clearly divides the pores, fissures and matrix parts of the coal samples. The pores in the coal samples are nonuniformly distributed and vertically connected, and very small pores exist in isolation. An accurate pore model is of great significance for the analysis of fluid seepage in coal samples.

(2) The pore radius, pore throat length and pore connectivity parameters of the coal samples are analysed by using the maximum sphere method. The coal samples contain 3458 pore points, 151 pore throat channels, a volume of 3.6735×1013 μm^3^, and a pore capacity of 9.098×1010 μm^2^. A pore space analysis indicates that the coal samples contain micropores (79.92%) and transition pores (17.29%), the pore volume ratio of which is 11.99%; when including the moderately developed mesopores (2.79%), the pore volume ratio is 88.01%. The coal samples exhibit favourable three-dimensional space constants. The mesopores that connect the pores provide the main seepage channels, and the contribution of isolated pores to the percolation capacity is negligible.

(3) The seepage simulation results based on the real pore-fracture network model show that the seepage region of fluid in the coal seam is divided into low-, medium- and high-speed regions. The morphological characteristics and connectivity of the seepage channels determine the permeability. The more connectivity there is, the larger the pore radii and the more uniform the morphological properties; thus, the more connectivity there is, the higher the permeability. The fluid seepage in the coal seam is driven by the following factors: pore connectivity compactness > pore throat channel dimensions > pore throat channel structure distribution characteristics.

(4) The pore distribution characteristics of the studied coal seam are analysed qualitatively, and the three-dimensional pore-fracture network structure is characterized quantitatively. From the microscopic point of view, the migration trajectory of fluid in the microscale seepage channels is analysed. The results suggest that this proposed method for evaluating the physical properties and flow capacity of coal seams is accurate.

## Supporting information

S1 File (RAR)
